# Flucloxacillin decreases tacrolimus blood trough levels: a single-center retrospective cohort study

**DOI:** 10.1007/s00228-020-02968-z

**Published:** 2020-07-25

**Authors:** Herman Veenhof, Hugo M. Schouw, Martine T. P. Besouw, Daan J. Touw, Valentina Gracchi

**Affiliations:** 1 Department of Clinical Pharmacy and Pharmacology, University Medical Center Groningen, University of Groningen, PO Box 30.001, 9700 RB Groningen, The Netherlands; 2 Department of Pediatric Nephrology, Beatrix Children’s Hospital, University Medical Center Groningen, University of Groningen, Groningen, The Netherlands; 3 Department of Pharmaceutical Analysis, Groningen Research Institute of Pharmacy, University of Groningen, Groningen, The Netherlands

**Keywords:** Tacrolimus, Everolimus, Flucloxacillin, CYP3A4, Drug-drug interaction, Blood trough levels, Therapeutic drug monitoring

## Abstract

**Purpose:**

Tacrolimus and everolimus are widely used to prevent allograft rejection. Both are metabolized by the hepatic cytochrome P450 (CYP) enzyme CYP3A4 and are substrate for P-glycoprotein (P-gp). Drugs influencing the activity or expression of CYP enzymes and P-gp can cause clinically relevant changes in the metabolism of immunosuppressants. Several case reports have reported that flucloxacillin appeared to decrease levels of drugs metabolized by CYP3A4 and P-gp. The magnitude of this decrease has not been reported yet.

**Methods:**

In this single-center retrospective cohort study, we compared the tacrolimus and everolimus blood trough levels (corrected for the dose) before, during, and after flucloxacillin treatment in eleven transplant patients (tacrolimus *n* = 11 patients, everolimus *n* = 1 patient, flucloxacillin *n* = 11 patients).

**Results:**

The median tacrolimus blood trough level decreased by 37.5% (interquartile range, IQR 26.4–49.7%) during flucloxacillin treatment. After discontinuation of flucloxacillin, the tacrolimus blood trough levels increased by a median of 33.7% (IQR 22.5–51.4%). A Wilcoxon signed-rank test showed statistically significantly lower tacrolimus trough levels during treatment with flucloxacillin compared with before (*p* = 0.009) and after flucloxacillin treatment (*p* = 0.010). In the only available case with concomitant everolimus and flucloxacillin treatment, the same pattern was observed.

**Conclusions:**

Flucloxacillin decreases tacrolimus trough levels, possibly through a CYP3A4 and/or P-gp-inducing effect. It is strongly recommended to closely monitor tacrolimus and everolimus trough levels during flucloxacillin treatment and up to 2 weeks after discontinuation of flucloxacillin.

**Electronic supplementary material:**

The online version of this article (10.1007/s00228-020-02968-z) contains supplementary material, which is available to authorized users.

## Introduction

The calcineurin inhibitor tacrolimus and the mammalian target of rapamycin (mTOR) inhibitor everolimus are widely used in organ transplantation to prevent allograft rejection [1]. Because both immunosuppressants have a narrow therapeutic window and show great inter- and intraindividual variability in pharmacokinetics, frequent monitoring of blood drug levels is required [2]. Tacrolimus and everolimus are primarily metabolized by hepatic cytochrome P450 (CYP) enzymes and are substrates for P-glycoprotein (P-gp) [2–4]. Drugs influencing the activity or expression of CYP enzymes or P-gp can cause clinically relevant changes in immunosuppressant metabolism resulting in toxic or subtherapeutic tacrolimus and everolimus blood levels [5].

Due to immunosuppressants, transplant patients are at increased risk for bacterial infections and flucloxacillin is a relatively frequently used antibiotic. This small-spectrum antibiotic of the isoxazolyl-penicillin type is used to treat Gram-positive bacterial infections. Although CYP3A4 is involved in the metabolism of flucloxacillin, the drug itself does not show inhibition of CYP P450 isoenzymes, as shown by in vitro assays [6–8]. However, several cases have been reported in which flucloxacillin appeared to decrease levels of drugs metabolized by CYP3A4, such as voriconazole, cyclosporin A, and quinidine [8–11]. These findings suggest that flucloxacillin might be a CYP3A4 inducer. In fact, all clinically relevant doses of flucloxacillin appear to induce CYP3A4 and P-gp through the activation of nuclear PXR (Pregnane X Receptor) [8].

Recently, we observed subtherapeutic trough levels of both everolimus and tacrolimus during flucloxacillin treatment in an 11-year-old transplant patient. At the age of 18 months, he had received a living-related combined kidney and liver transplant because of primary hyperoxaluria type 1. He was treated with prednisolone, everolimus, and tacrolimus to prevent allograft rejection. After placement of a nephrostoma at the age of 11, he presented at the outpatient clinic with recurrent episodes of *Staphylococcus aureus* pyelonephritis. Based on antibiogram results, he was treated with flucloxacillin. After starting flucloxacillin, trough levels of everolimus and tacrolimus decreased from 5.3 to 1.5 μg/L and from 2.9 to 2.3 μg/L, respectively, despite a dose increase of 1 mg/day for everolimus and 1 mg/day for tacrolimus. After discontinuation of flucloxacillin, the trough levels of everolimus and tacrolimus increased spontaneously within 3 days. Seven days later, trough levels increased even further, reaching values of 8.1 μg/L for everolimus and 7.1 μg/L for tacrolimus (target trough levels for everolimus and tacrolimus were 4–6 μg/L and 3–5 μg/L, respectively). A comprehensive PubMed database search for drug-drug interaction between flucloxacillin and tacrolimus or everolimus led to no results. Later, while conducting this study, we found a case series of 4 adult heart transplant patients reporting a decrease in tacrolimus (*n* = 4) and everolimus (*n* = 1) blood trough levels during flucloxacillin treatment [12]. In their study, Gellatly et al. hypothesize that this is caused by the CYP3A4-inducing properties of flucloxacillin [12].

We hypothesized that the changes in tacrolimus and everolimus trough concentration in our case were caused by induction of CYP3A4 by flucloxacillin. In order to support this hypothesis and to provide additional evidence of this potential drug-drug interaction, we conducted a single-center retrospective cohort study in which the effect of flucloxacillin on whole blood trough levels of tacrolimus and everolimus was assessed and quantified.

## Methods

### Patient selection

This single-center retrospective cohort study was performed at the University Medical Center Groningen (UMCG), The Netherlands. In this hospital, the following numbers of transplantations were performed in 2018: kidney *n* = 166, liver *n* = 73, lung *n* = 35, pancreas *n* = 9, heart *n* = 8, small bowel *n* = 2, and stem cell transplantations *n* = 183 [13].

We intended to include all solid organ and allogeneic stem cell transplantation patients who were treated with flucloxacillin while receiving immunosuppression with tacrolimus and/or everolimus in the period of between January and December 2018. Patients were identified using a query in the hospital electronic information system. Information on trough levels of tacrolimus and everolimus were obtained 1 year before, during, and up until 1 year after treatment with flucloxacillin. Due to the retrospective and descriptive nature of this study, the need to provide informed consent was waived by the UMCG medical ethics committee (METc 2019/199).

The following exclusion criteria were applied:

- Use of the following known CYP inhibitors/inducers as defined by the Dutch National Pharmacists Database: carbamazepine, efavirenz, enzalutamide, phenobarbital, phenytoin, hypericum, mitotane, nevirapine, primidone, rifabutin, rifampicin, cobicistat, caspofungin, etravirine, sevelamer, clarithromycin, cobicistat, erythromycin, itraconazole, ketoconazole, ritonavir, voriconazole, chloramphenicol, danezole, diltiazem, felodipine, fluconazole, nifedipine, posaconazole, imatinib, and grapefruit juice [14]
- Decreased liver function, increased transaminases (ALAT > 50 U/L, ASAT > 45 U/L), or enteritis/colitis during treatment with flucloxacillin, since significant CYP450 metabolism takes place in liver and intestine [3, 15]
- Patients younger than 1 year of age, due to immature CYP450 function in infants [16]

### Clinical characteristics

For each patient, data regarding gender, age, transplant type, transplant date, body mass index (BMI), blood-drug levels, and corresponding doses of tacrolimus and everolimus were collected from the electronic health records (EHR). All tacrolimus and everolimus whole blood trough levels were determined as part of routine care by validated LC-MS/MS analysis at the UMCG [17].

### Outcomes

Because immunosuppressants show great interindividual variability in blood trough levels, trough levels were normalized for the dose by dividing the trough level (μg/L) by the dose (mg), giving the unit trough level/dose (μg/L/mg). This method allows comparison between patients [18].

The following indicators were determined to quantify the effect of flucloxacillin on tacrolimus trough levels [19].

1. The number of patients with a decrease in tacrolimus or everolimus trough levels during flucloxacillin treatment. A decrease was defined as a lower mean whole blood trough level/dose during flucloxacillin treatment compared with a maximum period of 1 year before start of flucloxacillin treatment.
2. The number of patients with an increase in tacrolimus or everolimus whole blood trough levels after flucloxacillin treatment. An increase was defined as a higher mean whole blood trough level/dose for a period up to 1 year after flucloxacillin treatment, compared with during flucloxacillin treatment.
3. The tacrolimus or everolimus mean trough level before, during and after treatment with flucloxacillin, corrected for the tacrolimus or everolimus dose. The mean blood trough levels/dose before flucloxacillin treatment consists of the mean of the available tacrolimus or everolimus trough levels up to 1 year before flucloxacillin treatment, divided by the dose at time of trough concentration sampling. The mean blood trough levels/dose during flucloxacillin treatment consists of all available tacrolimus or everolimus trough levels during flucloxacillin treatment starting from the day after start of flucloxacillin treatment until the last day of flucloxacillin treatment. The mean tacrolimus or everolimus trough levels/dose after flucloxacillin treatment consists of all the available trough levels divided by dose starting from 1 day after discontinuation of flucloxacillin up to 1 year after discontinuation of flucloxacillin.
4. Median tacrolimus or everolimus blood trough levels, corrected for the dose, before, during, and after treatment are calculated in a comparable manner to the description above.
5. The mean and median before/during flucloxacillin ratios, calculated by dividing the median (or, respectively, the mean) of tacrolimus and everolimus blood trough level/dose before flucloxacillin treatment by the median (or, respectively, the mean) of blood trough level/dose after flucloxacillin treatment. These values are used to quantify the change in tacrolimus or everolimus blood trough levels/dose. The same ratios were calculated during and after flucloxacillin treatment.
6. The median ratios were converted to a percentage change of tacrolimus or everolimus whole blood levels. The median percentages are given with an interquartile range (IQR).

### Statistical analysis

Mean and median tacrolimus or everolimus whole blood trough levels divided by the immunosuppressant dose before, during, and after flucloxacillin treatment were calculated and compared. The normality of the distribution of the immunosuppressant blood trough levels divided by the dose, per group, was tested by conducting a Shapiro-Wilk normality test. Statistical significance was set at *p* < 0.05. Based on the type of distribution and the sample size, a non-parametric Wilcoxon signed-rank test and/or a parametric paired *t* test were performed to compare the mean values of the groups (*p* < 0.05) [19]. The distribution of each group was plotted in a box plot to allow visual comparison of median values and the overall values. All statistical analyses were performed with IBM SPSS statistics version 23 (Armonk, NY: IBM Corp, USA). All categorical data were expressed as percentages. Numeric data were expressed as mean ± standard deviation (SD) or as median with min-max and IQR, as appropriate.

## Results

Eleven patients, including the case described in the “Introduction” section, were identified by applying the predefined criteria. All included patients used tacrolimus. Apart from the index patient, no other patient was treated with everolimus. The patient characteristics are shown in Table 1. One subject (patient 6) had no available data on tacrolimus trough levels before flucloxacillin use. Data were complete for ten patients. In nine of the eleven patients, there was a decrease in tacrolimus trough levels during flucloxacillin treatment. After discontinuing flucloxacillin, ten patients showed an increase in tacrolimus trough levels. Data on individual patients are shown in Online Resource 1.

**Table 1:** Patient characteristics

Patient	Gender	Age (years)	Body weight (kg)	BMI (kg/m^2^)	Transplant type	Time from transplantation at the moment of starting with flucloxacillin treatment
1	Male	26	71	20.5	Lung	9 days
2	Male	39	84	25.9	Liver	14 days
3	Male	71	77	26.0	Allogeneic stem cell	5 years, 6 months, 12 days
4	Male	38	75	22.4	Lung	5 years, 3 months, 2 days
5	Male	38	93	29.5	Lung	7 years, 6 months, 25 days
6	Male	6	21	15.5	Liver	4 days
7	Male	4	17	14.9	Liver	2 years, 9 months, 26 days
8	Male	72	78	25.5	Kidney	1 day
9	Female	64	49	19.6	Lung	10 years, 4 months, 10 days
10	Female	48	60	23.7	Liver	111 days
11	Male	11	26	13.4	Kidney and liver	10 years, 0 months, 20 days

**Fig. 1:**
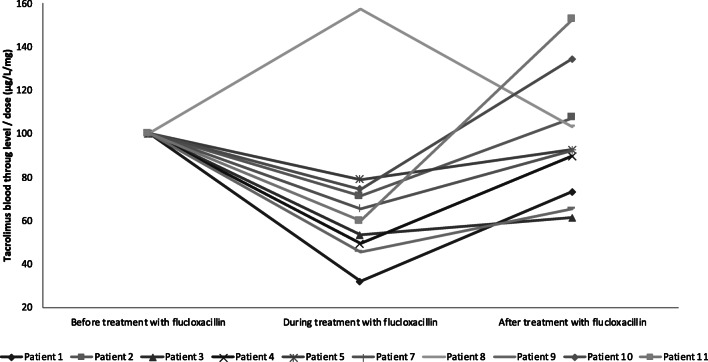
Changes in tacrolimus blood trough levels corrected for the dose. The blood trough level/dose value before the treatment with flucloxacillin is set at 100%. Changes in tacrolimus blood trough levels/dose during and after flucloxacillin treatment are given as a percentage of this value. Because patient 6 had no data before the flucloxacillin treatment, no data can be shown

The mean tacrolimus blood trough level corrected for the dose before flucloxacillin was 2.3 μg/L/mg (± 1.6), during treatment 1.3 μg/L/mg (± 1.0), and after treatment 1.9 μg/L/mg (± 1.4). The median tacrolimus blood trough level dropped by 37.5% (IQR 26.4–49.7%) during flucloxacillin treatment. After discontinuation of flucloxacillin treatment, the median tacrolimus blood trough level increased by 33.7% (IQR 22.5–51.4%). Table 2 shows the values of the measured outcomes. Online Resource 2 shows a box plot of these data. Changes in tacrolimus blood trough levels/dose are shown in Fig. 1. All but one patient (patient 8) showed a lower tacrolimus trough level divided by dose during flucloxacillin treatment.

**Table 2:** Measured outcomes for comparing tacrolimus trough levels before, during, and after flucloxacillin treatment

Parameter	Before flucloxacillin	During flucloxacillin	After flucloxacillin
Mean blood trough level/dose, μg/L/mg (± SD)	2.3 (± 1.6)* *n* = 10	1.3 (± 1.0)	1.9 (± 1.3)
Median blood trough level/dose, μg/L/mg(min-max)	2.3 (0.52–3.77)	1.3 (0.3–3.3)	1.5 (0.5–3.8)
Median blood trough level/dose ratios before/during and after/during flucloxacillin (min-max)	–	1.6 (0.6–3.1)	1.7 (0.7–3.0)
Median blood trough level/dose ratios before/during and after/during flucloxacillin, percentage (IQR)	–	− 37.5 (26.4–49.7)	+ 33.7 (22.5–51.4)
Mean ratio before/during and after/during flucloxacillin treatment (± SD)	–	1.7 (± 0.7)	1.5 (± 0.7)

*n = 11 unless otherwise specified. The mean/median blood trough levels/dose before flucloxacillin treatment consists of the mean/median of the available tacrolimus trough levels up to 1 year before flucloxacillin treatment, divided by the dose at time of trough concentration sampling. The mean/median blood trough levels/dose during flucloxacillin treatment consists of all available tacrolimus trough levels during flucloxacillin treatment starting from the day after start of flucloxacillin treatment until the last day of flucloxacillin treatment. The mean/median tacrolimus trough levels/dose after flucloxacillin treatment consists of all the available trough levels divided by dose starting from 1 day after discontinuation of flucloxacillin up to 1 year after discontinuation of flucloxacillin

The Shapiro-Wilk test of normality indicated that all three groups had a normal distribution (*p* > 0.05) of mean blood trough levels (before flucloxacillin *p* = 0.32, during flucloxacillin *p* = 0.39, and after flucloxacillin *p* = 0.29). Mean blood trough levels/dose before, during, and after flucloxacillin use was compared with both a paired *t* test and a non-parametric test because of the small sample size.

Both tests showed a significant difference in mean tacrolimus blood trough levels corrected for dose before and during flucloxacillin treatment (paired *t* test: *p* = 0.006, Wilcoxon: *p* = 0.009); a significant difference between, during, and after flucloxacillin treatment (paired *t* test: *p* = 0.003, Wilcoxon: *p* = 0.010); but no significant difference between before flucloxacillin treatment and after flucloxacillin treatment (paired *t* test: *p* = 0.179, Wilcoxon: *p* = 0.262).

## Discussion

In this retrospective, single-center study, we showed that treatment with flucloxacillin was associated with a median decrease by 37.5% of tacrolimus trough levels. Moreover, the mean tacrolimus blood trough levels corrected for the dose before and during flucloxacillin treatment were statistically significantly different, as well as the mean tacrolimus blood trough levels corrected for the dose during and after flucloxacillin treatment. These changes in trough levels can be of great importance in the clinical management of transplant patients, as unintentional and unnoticed low tacrolimus levels represent a risk for allograft rejection.

Our findings are in line with the findings of the case series by Gellatly et al. [12], showing a decrease of tacrolimus trough levels during flucloxacillin treatment in 4 adult heart transplant patients. The additional value of our study is that we were able to quantify the change in trough levels by correcting the tacrolimus blood trough levels for the prescribed dose. Moreover, our results show that the reported changes are detectable in different types of organ transplants and throughout different age groups.

Nevertheless, the study from Muilwijk et al. reported different results, stating that flucloxacillin did not decrease tacrolimus trough levels [10]. However, it has to be kept in mind that the study from Muilwijk et al. focused on investigating the drug-drug interaction between flucloxacillin and voriconazole, which is a CYP3A4 inhibitor. This might mitigate the potential CYP3A4-inducing effect of flucloxacillin. No details on tacrolimus doses and trough levels were provided [10]. Moreover, the potential effect of flucloxacillin on tacrolimus might have been overlooked in their study since, in general, frequent dose adjustments occur on the base of frequent trough level control. This problem can be overcome by correcting the trough levels for the dose, as done in our study.

The induction of CYP3A4 has been described for several drugs, including flucloxacillin [8]. It is important to note that the maximal effect of CYP3A4 induction is achieved over a period of 10–14 days [20]. After discontinuation of a CYP3A4 inducer, the time needed to restore the basal level of metabolism is usually 10–14 days [20]. This delay is also reported in a case where levels of quinidine decreased during flucloxacillin therapy and increased again after discontinuing flucloxacillin [9]. In our retrospective study, a similar delay was seen. Moreover, the magnitude of the effect was comparable with the effect observed in a report on cyclosporin A levels after treatment with flucloxacillin, where the mean cyclosporin A levels fell by 55% within 3 days in seven adult kidney transplant patients [11]. In addition, similar decrease in both tacrolimus and everolimus trough levels has been reported during rifampicin therapy [21–23].

A possible mechanism behind the drug-drug interaction between flucloxacillin and tacrolimus might be that flucloxacillin activates the nuclear hormone Pregnane X Receptor (PXR), which induces the expression of both CYP3A4 and P-gp [8, 11, 20]. This hypothesis is supported by a recent study in human hepatocytes where flucloxacillin and dicloxacillin induced CYP3A4 enzymes through the same PXR mechanism [24]. It is hypothesized that induction of CYP3A4 does not always have the same effect on drug metabolism as induction of P-gp [25]. This is shown in a study where CYP3A4, but not P-gp, restricted oral bioavailability of everolimus in mice despite everolimus being a substrate for P-gp [4, 26]. However, a study investigating the role of the potent CYP3A4- and P-gp inducer rifampicin showed similar effects on both CYP3A4 and P-gp and drugs metabolized by either P-gp, CYP3A4, or both P-gp and CYP3A4 [23]. Since PXR induces both CYP3A4 and P-gp, it is unknown if the decrease in tacrolimus (and probably everolimus) trough levels observed in this study is a result of either CYP3A4 induction, P-gp induction, or a combination of both [4, 26, 27]. In addition, there may also be other explanations for the observed lower trough levels of tacrolimus. Unknown factors such as P-gp pharmacogenetics might be of influence. In our study, all patients except for one showed the same pattern of tacrolimus decrease during flucloxacillin therapy. Only patient 8 showed an increase in mean tacrolimus blood trough levels corrected for the dose during and after flucloxacillin treatment. The EHR of patient 8 mentioned, during flucloxacillin treatment, alternating episodes of obstipation and laxative-induced diarrhea, which might have played a role. Although it is known that diarrhea can lead to increased tacrolimus exposure, it is unknown if laxative-induced diarrhea can cause this same phenomenon [28]. Therefore, we chose not to exclude this patient from our analysis. However, if this patient were to be excluded, the effect of flucloxacillin would even be greater, with a median decrease of 40.2% in tacrolimus trough concentration during flucloxacillin treatment. Further in vivo pharmacokinetic studies are needed to confirm the hypothesis of a drug-drug interaction between flucloxacillin and tacrolimus and assess the extent of CYP3A4 and/or P-gp induction by flucloxacillin.

We have also considered other possible drug-drug interactions. Among others, we have considered an interaction with steroid drugs. In our study, nine out of eleven patients used steroids and some had dose changes during the study period (data not shown). It has been previously hypothesized that steroids such as prednisolone can increase the tacrolimus dose requirement [12]. However, literature considering this effect is inconsistent and mentions that the interaction might only be relevant in specific CYP3A5 genotypes [29, 30]. Therefore, we conclude, in accordance with Gellatly et al., that the potential impact of steroid use is minimal, if at all present [12].

Our retrospective cohort study has several limitations. A first limitation is the low number of patients included in the study (*n* = 11) as this increases the risk of bias. Despite the low number and diversity of patients in this study with regard to both age and transplant type, we were nevertheless able to show a relationship between therapy with flucloxacillin and a decrease in tacrolimus blood levels. However, a larger study is needed to confirm our results.

A second limitation is that only retrospective data from the EHR were available. Certainly, a prospective pharmacokinetic cross-over study would offer a more controlled approach for the assessment of the interaction between flucloxacillin and tacrolimus. In such a study, the effect of the potential drug-drug interaction could be measured in healthy volunteers by comparing tacrolimus area under the curve (AUC) before, during, and after flucloxacillin treatment.

Another limitation of this study is that we were not able to gather enough information about the relationship between everolimus trough levels and flucloxacillin therapy since only one patient was treated with flucloxacillin while on everolimus. Based on the case described in the Introduction section (see also Online Resource 1, patient 11), and on the fact that everolimus is also metabolized by CYP3A4, a similar effect of flucloxacillin on everolimus trough levels can be expected. The effect on everolimus could even be more profound because everolimus is metabolized primarily by CYP3A4, while tacrolimus is metabolized by both CYP3A4 and CYP3A5 [3]. However, this hypothesis can only be confirmed in larger studies.

In conclusion, we reported changes in tacrolimus and everolimus trough levels during flucloxacillin therapy. Until our observations are complemented by additional studies on this subject, we advise physicians and pharmacists to be aware of a possible drug-drug interaction. Close monitoring of immunosuppressant trough levels during flucloxacillin treatment and up to at least 2 weeks after discontinuation of flucloxacillin is strongly recommended.

## Electronic supplementary material

ESM 1 (DOCX 72 kb).

ESM 2 (DOCX 61 kb).

## Data Availability

The datasets generated during and analyzed during the current study are available from the corresponding author on a reasonable request.
