# Supplementary material for: Flucloxacillin decreases tacrolimus blood trough levels: a single-center retrospective cohort study
Source: Eur J Clin Pharmacol. 2020 Jul 25;76(12):1667–73. doi: 10.1007/s00228-020-02968-z (PMC7661399; doi:10.1007/s00228-020-02968-z)
Supplement: Supplementary file 1 — (DOCX 72 kb). [file 228_2020_2968_MOESM1_ESM.docx]

**Online Resource 1**

Article title:
Flucloxacillin decreases tacrolimus blood trough levels: a single-center retrospective cohort study

Journal name:
European Journal of Clinical Pharmacology

Author names:
Herman Veenhof^1^, Hugo M. Schouw, Martine T.P. Besouw, Daan J. Touw, Valentina Gracchi

Afiliation
1 University of Groningen, Department of Clinical Pharmacy and Pharmacology, University Medical Center Groningen, Groningen, The Netherlands.

h.veenhof@umcg.nl

**Online Resource 1 – Individual patient details**

This supplement shows the individual data per patient as graphs. For each patient two graphs are shown. The first graph shows the tacrolimus trough concentration corrected for the dose before, during and after flucloxacillin therapy. The second graphs shows the tacrolimus trough concentrations in µg/L and tacrolimus dose in mg at the time the blood sample was drawn before, during and after flucloxacillin therapy. For patient 11, information on everolimus use is also shown in the graph.
