# Supplementary material for: Flucloxacillin decreases tacrolimus blood trough levels: a single-center retrospective cohort study
Source: Eur J Clin Pharmacol. 2020 Jul 25;76(12):1667–73. doi: 10.1007/s00228-020-02968-z (PMC7661399; doi:10.1007/s00228-020-02968-z)
Supplement: Supplementary file 2 — (DOCX 61 kb). [file 228_2020_2968_MOESM2_ESM.docx]

**Online Resource 2**

Article title:
Flucloxacillin decreases tacrolimus blood trough levels: a single-center retrospective cohort study

Journal name:
European Journal of Clinical Pharmacology

Author names:
Herman Veenhof^1^, Hugo M. Schouw, Martine T.P. Besouw, Daan J. Touw, Valentina Gracchi

Afiliation
1 University of Groningen, Department of Clinical Pharmacy and Pharmacology, University Medical Center Groningen, Groningen, The Netherlands.

h.veenhof@umcg.nl

**Online Resource 2 - Boxplot**

Boxplot showing distributions of tacrolimus blood trough levels divided by dose before, during and after flucloxacillin. The mean/median levels per patient consist of all available tacrolimus blood trough levels/dose before to (up until 1 year), during and after flucloxacillin. The black lines represent the median blood trough levels/dose and the dots represent the mean blood trough levels/dose. The boxes show the values between the 25th and 75th percentile of the median value. The whiskers represent the min-max values of the median for each group.
